# Trapezius activity of fibromyalgia patients is enhanced in stressful situations, but is similar to healthy controls in a quiet naturalistic setting: a case-control study

**DOI:** 10.1186/1471-2474-14-97

**Published:** 2013-03-18

**Authors:** Rolf Harald Westgaard, Paul Jarle Mork, Håvard Wuttudal Lorås, Roberto Riva, Ulf Lundberg

**Affiliations:** 1 Department of Industrial Economics and Technology Management, Norwegian University of Science and Technology, Trondheim, Norway; 2 Department of Human Movement Science, Norwegian University of Science and Technology, Trondheim, Norway; 3 Department of Psychology, Stockholm University, Stockholm, Sweden; 4 CHESS (Centre for Health Equity Studies), Stockholm University, Stockholm, Sweden

**Keywords:** Stress, Heart rate, Surface electromyography, Sympathetic activity

## Abstract

**Background:**

Muscle activity and pain development of fibromyalgia (FM) patients in response to mental stress show inconsistent results, when compared to healthy controls (HCs). A possible reason for the inconsistent results is the large variation in stress exposures in different studies. This study compares muscle responses of FM patients and HCs for different modes and levels of imposed stress, to elucidate features in stress exposures that distinguish stress responses of FM patients from HCs.

**Methods:**

Upper trapezius (clavicular and acromial fibers), deltoid, and biceps surface electromyographic (sEMG) activity was recorded in FM patients (n=26) and HCs (n=25). Heart rate (HR) was recorded and used as indicator of autonomic activation. Tests included inspiratory breath holding (sympathetic activation procedure), mental stress tests (color-word test and backward counting; 28 min), instructed rest prior to stress test (30 min TV watching), and controlled arm movement. sEMG and HR was also recorded during an unrestrained evening stay at a patient hotel. The 5-min period with lowest trapezius muscle activity was determined. Pain (shoulder/neck, low back pain) and perceived tension were scored on VAS scales at the start and the end of the stress test and at bedtime.

**Results:**

Trapezius sEMG responses of FM patients were significantly higher than HCs during sympathetic activation, mental stress, and instructed rest, but similar during arm movement and unrestrained evening activity. HR of FM patients and HCs was similar during mental stress and in the evening, including the 5-min period with lowest trapezius activity. Muscle activity of FM patients during the stress test (with shoulder/neck pain development) and the evening stay (no pain development) was similar.

**Conclusions:**

FM patients show elevated muscle activity (in particular trapezius activity) in situations with imposed stress, including sympathetic activation, and putative anticipatory stress. Muscle activity and HR were similar to HCs in instructed arm movement and in a situation approaching low-stress daily living. Pain development of FM patients during the stress test may be due to activation of several stress-associated physiological systems, and not obviously caused by muscle activity in isolation.

## Background

The central nervous system (CNS) is clearly implicated in the pathophysiology of fibromyalgia (FM) [1-6]; however, a peripheral contribution is likely since muscle tender points are integral to the diagnosis [7-9]. Muscle fiber pathology of FM patients has been reported [10]. Consequently, studies have investigated the association between FM and muscle overexertion or motor control features indicating overexertion, but with mixed results [11-14]. Another line of investigation has focused on muscle activity with stress exposure. Many studies have shown muscle activity in response to imposed stress, with trapezius among the most responsive in this respect [15]. A working hypothesis is that FM patients, due to dysregulation of the autonomic system [16-18], may generate more muscle activity and thereby pain in response to stressful influences. Studies in our laboratory have provided results that both support [19] and fail to support [20] this hypothesis. It may further be questionned whether elevated, but low levels of muscle activity in FM patients relative to healthy controls (HC), observed under strictly controlled laboratory conditions are replicated in situations close to normal living. A challenge with respect to the latter query is to establish a condition whereby habitual, unrestrained activities of the two groups are comparable.

Differences in muscle responses of FM patients to stress exposure may be due to inadvertent differences in conditions in which the experimental stress test was presented. A test condition with relatively mild stress exposure was used in both of the previous studies [19,20], but circumstances such as laboratory environment and instructions to the subjects differed, which may influence responses [21]. Differential responses to stress exposure may depend on the mode and intensity of the stressful experience. Further, heterogeneity of the FM patient population is shown [22-24] and may account for differences in responses in studies based on limited material.

The present study was carried out in the setting of a hospital patient hotel, where subjects stayed overnight. FM patients and HCs were not allowed to leave the hotel, but were otherwise free to move around. This allowed a realistic comparison of upper trapezius activity during unrestrained sedentary conditions, supplementing a sequence of laboratory tests carried out when subjects arrived in the afternoon: tests with relatively high stress exposure, with sympathetic provocation, a test promoting relaxation with minimal body movement, and a test with controlled arm movement.

It was hypothesized that upper trapezius muscle activity is enhanced for FM patients relative to HCs in situations perceived stressful, potentially dependent on stress level and mode, but is similar in low-stress vocational living. The study aimed to examine this hypothesis by comparing trapezius activity of FM patients and HCs in the above listed test conditions and in sedentary living at the patient hotel, including quiet seated activity and an evening meal. In an earlier study, stress-associated trapezius activity was enhanced in the clavicular direction of the muscle, relative to activity in shoulder elevation [25]. The standardized upper trapezius electrode location [26] was therefore supplemented with a second trapezius electrode placed in a clavicular position. Heart rate (HR) was recorded as a marker of an activation response and to indicate autonomic system balance by HR variability (HRV) measures. Deltoid and biceps were included as examples of muscles with lower responses to stress [15]. Pain in shoulder/neck and in low back, and perceived general tension were scored on visual analogue scales (VAS) [27-29]. Subjectively scored variables were used to elucidate any effect of putative heterogeneity of FM patients [24].

## Methods

### Participants

Twenty-six female patients with FM and 25 age-matched (±3 years) female HCs participated in the study (Table 1). The patients were mainly recruited through the local FM association while HCs were recruited among donors to the hospital blood bank. Inclusion criteria were age between 35 and 64 years. Upon inclusion to the study, eligible FM patients underwent a clinical examination to verify the FM diagnosis as defined by the American College of Rheumatology [30]. Number of years since first symptoms and number of years since confirmed diagnosis were retrieved from each participant’s medical record. Patients were excluded if they had: a) cardiorespiratory, cerebrovascular, neurologic, neuromuscular, endocrine, infectious, metabolic, lung, or cancer disease, b) injury that affected function, c) connective tissue disorder, d) tendinitis or capsular affection of the shoulder joint, or e) high blood-pressure (i.e., systolic pressure >140 mmHg or diastolic pressure >90 mmHg) or were taking anti-hypertensive medication. Participants were also excluded if they were taking medication that may interact with neural, vascular, or muscular function or the physiological measurements to be performed (e.g., antidepressants, antiepileptics, β-blockers). FM patients that used analgesics and/or sleep medicine on a regular basis were instructed to cease medication two days prior to the experiment. The study protocol was approved by the Regional Committee for Ethics in medical research (project no. 4.2005.2728) and all participants signed an informed consent before inclusion. The study was carried out in the premises of a hotel used by outpatients, with easy access to university laboratories. It is part of a comprehensive study including sleep recordings and the collection of samples for cortisol and catecholamine analyses [31-34].

**Table 1:** Subject characteristics

	**FM patients**	**Controls**
	**Mean (SD), range**	**Mean (SD), range**
Age (years)	51.8 (8.5), 38-64	52.4 (8.7), 37-64
Body mass index (kg/m^2^)	26.7 (5.3), 17.8-38.9	24.6 (3.3), 20.5-35.0
No. of tender points	15.7 (2.2), 11-18	Not Addressed
Years since diagnosis	5.5 (6.3), 0-26	Not Addressed
Years since first symptoms	11.6 (6.8), 3-33	Not Addressed
	% (n)	% (n)
Employment fraction ≥50%	27 (7)	88 (22)
Smokers	27 (7)	24 (6)
Exercise (≥1 session per week)	96 (25)	88 (22)

Descriptive statistics for demographic and other background variables of the fibromyalgia (FM) patients and the healthy controls.

### Procedure

**Figure 1:**
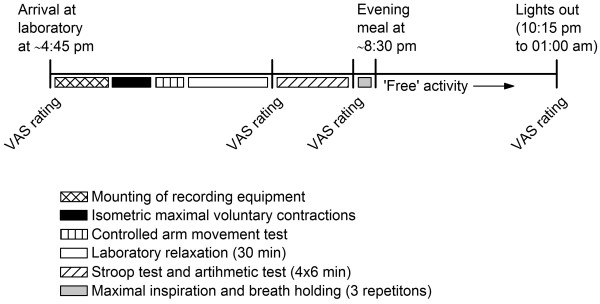
**Order and time schedule for the data collection.** Recording equipment was mounted immediately after arrival at the laboratory (~4:45 pm, range 4:30–5:00 pm) followed by an experimental session with laboratory recordings. After an evening meal at ~8:30 pm the subjects were free to choose activity until bedtime. sEMG activity (trapezius, deltoideus, and biceps) and heart rate were recorded continuously from start of the experimental session until bedtime. Pain (shoulder/neck, low back), general tension, and perceived stress were scored on Visual Analogue Scale (VAS) at four occasions.

The order and time schedule for data collection is shown in Figure 1. Participants met in the laboratory at around 4.45 pm. After mounting the electrophysiological recording equipment, a session consisting of isometric maximal voluntary contractions (MVCs) and four test conditions was performed (Figure 1): a) controlled arm movement test (see below), b) Laboratory relaxation: participants were comfortably seated in an arm chair and watched a cartoon movie for 30 min, c) mental stress: four 6-min periods alternating between the Stroop test [35] and an arithmetic test with backward counting (mean duration 28 min, range 26–29 min), and d) activation of the sympathetic system during standing: intrathoracic pressure was increased by maximal inspiration and breath holding with epiglottis closed for ~15 s [36,37].

In the controlled arm movement test the participants moved either the dominant or non-dominant hand continuously, holding a pen, between three circles forming an equilateral triangle on a horizontal table [38]. The dominant hand moved in clockwise direction and the non-dominant hand in anti-clockwise direction. Circle diameter was 70 mm in the first trial and 4 mm in the second trial. Each trial lasted 2 min and a metronome paced the arm movement at 88 beats/min, i.e., participants were required to set a mark within the circles following the beat of the metronome. In Laboratory relaxation, participants were instructed to relax to the best of their ability. The experimenter that mounted the recording equipment and performed the tests was blinded to the diagnosis of the participants.

After the laboratory session the participants had an evening meal (bread, salad, fruits) at around 8.30 pm and were thereafter free to choose activity (e.g., reading, watching TV, playing solitaire with cards) but were instructed to stay inside the hotel. The participants filled in a short diary with description of evening activities and time period for each activity. Physiological responses were determined for “quiet seated activity” (reading or watching TV), for the evening meal, and for the 5-min period with lowest surface electromyographic (sEMG) activity of upper trapezius.

The participants scored pain level (shoulder/neck, low back), perceived general tension, and perceived stress on a VAS (0–100 mm) upon arrival in the laboratory, after Laboratory relaxation, after the mental stress test, and at bedtime (Figure 1). Pain intensity was scored after first indicating whether they at all felt pain (yes/no). All VAS scales were anchored by “very high” and “very low” at endpoints. Participants also scored their level of effort associated with the stress test and how stressful they perceived the test.

### Physiological recordings and analysis

A portable recording system (Myomonitor IV, Delsys Inc, Boston MA) was used to record force, sEMG, a modified lead II electrocardiogram (ECG), and respiratory frequency by a strain gauge embedded in a flexible belt placed around the chest just below the sternum.

For the ECG recordings, the QRS complex was detected, and the R-R intervals were derived on a beat-by-beat basis to determine heart rate (HR) during the stress test, during Laboratory relaxation in the laboratory, and during the 5-min evening period with lowest trapezius sEMG. Variables indicating heart rate variability (HRV) were determined [39,40]. Time domain measures included the standard deviation of the NN interval (SDNN) and the root mean square successive difference (RMSSD), based on 5-min periods of recordings. SDNN is thought to reflect total variability while RMSSD is mainly thought to reflect vagal modulation of heart activity. In case of the mental stress test and Laboratory relaxation in the laboratory, the second last 5-min period was selected. Frequency domain measures included the power of the low frequency component (LF; 0.04-0.15 Hz) and the high frequency component (HF; 0.15-0.4 Hz), as well as the ratio of the two components of the heart period power spectrum (LF/HF ratio). The recordings were visually inspected for ectopic heart beats and artifacts. Heart beats classified as non-normal beats were excluded from further analysis.

Bipolar sEMG (bar-shaped contact surfaces 1 by 10 mm, 10 mm spacing between surfaces) was recorded from the clavicular and acromial fibers of the dominant upper trapezius, middle deltoid, and biceps brachii. The midpoint of the acromial trapezius electrode was placed at a point 2 cm distal to the midpoint of a line from the spinous process of the 7^th^ cervical vertebra (C7) toward the lateral edge of the acromion [41,42]. The clavicular electrode was placed in parallel to the acromial electrode, 2 cm in the ventral (clavicular) direction [25]. For the middle deltoid the electrode was placed on a line from the acromion to the lateral epicondyle of the elbow, corresponding to the greatest bulge of the muscle. For the biceps brachii the electrode was placed on the line between the medial acromion and the fossa cubit at 1/3 from the fossa cubit. All signals were sampled at 1 kHz. The sEMG signal was band-pass filtered at 10–450 Hz and root-mean-square (RMS) values were calculated using a 100 ms non-overlapping window. Three isometric MVCs were performed for each muscle with simultaneous recording of sEMG and force. The highest sEMG response was used to normalize the sEMG signal (% EMG_max_). For each MVC, participants were instructed to develop maximal force within 1–2 s and thereafter hold the force for 3–5 s. A 1-min break was applied between each MVC. MVCs for trapezius and middle deltoid were performed with participants in an erect seated posture, with arms 90° abducted in the scapular plane. Resistance was applied just proximal to the elbow joint by adjustable straps connected to two strain-gauge force transducers secured to the floor. MVCs for the dominant biceps brachii were performed with participants in seated position, elbow flexed at 90°, and the elbow supported against the side of the body. Resistance was applied unilaterally to the dominant arm just proximal to the wrist joint by the adjustable strap that was connected to one of the force transducers. The force signal was low-pass filtered (10 Hz, Butterworth, 6^th^ order) and downsampled to 10 Hz before further analysis. Maximal force was determined as the median of the highest 0.5 s interval (i.e., median of 5 samples) for each MVC. Recordings of FM patients were inspected for indication of systematic force reduction with successive MVCs, but no such effect was found.

Table 2 presents maximal force and sEMG_max_ during MVCs. FM patients generated lower force than HC during shoulder abduction (not significant) and elbow flexion. This is an anticipated consequence of their patient status, but may represent a source of error if “true” force capacity of FM patients is masked by sub-maximal force generation in the calibration contractions due to, e.g., pain-associated inhibition. Correlation analyses showed no association of maximal force with indicators of present pain (pain in the laboratory, pain last 24 hrs, pain last week). Alternative sEMG calibrations were nevertheless explored by upward adjustment of patient sEMG_max_ values by 20% to compensate for group differences in maximal force. sEMG activity during the evening and in tests was quantified by median activity level and by rest time (sEMG amplitude <0.5% EMG_max_) [43].

**Table 2:** sEMG calibration contractions

	**FM patients**	**Controls**	**p**^**a**^
Force (N)			
Shoulder abduction	101 (37), 41-206	116 (21), 61-148	0.08
Elbow flexion	110 (29), 39-167	136 (27), 91-201	0.002
sEMG_max_ (μV)			
Clavicular trapezius	337 (189), 94-711	534 (203), 208-879	0.002
Acromial trapezius	276 (163), 87-615	501 (298), 119-1257	0.003
Middle deltoid	217 (96), 93-358	315 (105), 133-549	0.01
Biceps brachii	177 (81), 54-365	259 (131), 67-564	0.01

Maximal force and sEMG_max_ during isometric maximal voluntary contractions (i.e., shoulder abduction and elbow flexion) for fibromyalgia (FM) patients and healthy controls.

Values are mean (SD), range.

^a^Independent samples t-test.

### Questionnaires

Two questionnaires were administered to assess subjective health complaints and personality traits. The Subjective Health Complaints inventory consists of 29 questions concerning somatic and psychological complaints last 30 days [44]. For each item, severity of the complaint is rated on a 4-point scale (0=none, 3=severe) and duration by number of days during the last 30 days. The Karolinska Scales of Personality (KSP) is a self-rating questionnaire constructed to measure stable personality traits [45]. KSP consists of 135 items grouped into 15 subscales. Three subscales were used in this study: muscular tension, psychic anxiety, and somatic anxiety. Each item is scored on a 4-point Likert scale (0=does not apply at all, 3=applies completely).

Four indexes of potential relevance to influence physiological responses [24] were constructed from items in the two questionnaires: 1) musculoskeletal symptoms (pain in neck, shoulders and upper back, perceived stiffness in upper and lower body), 2) non-musculoskeletal symptoms (irregular HR, hot flushes, headache, abdominal pain, dizziness), 3) cognitive/psychological symptoms (anxiety, forgetfulness, depression), 4) muscle tension (tension in jaw muscles, feeling of tenseness when trying to sleep, difficulty in relaxing). The index scores (average of the included items) were considered long-term traits, to be distinguished from VAS scores of pain, stress, and perceived general tension on the experimental day [27].

### Statistical analysis

The Kolmogorov-Smirnov test was used to explore normality of dependent variables. All HR variables, all symptom indexes, most force variables (three of four), and most sEMG_max_ variables (six of eight) were normally distributed. None of the sEMG variables or VAS-score variables was normally distributed. The independent samples t-test was used to test differences between groups for normally distributed data while the Mann–Whitney U test was used to test group differences for non-normally distributed data. A mixed design repeated measures ANOVA was used to test differences between repeated recordings of HR and HRV. If Mauchly’s test indicated violation of sphericity, a Greenhouse-Geisser correction was applied [46]. Friedman’s ANOVA was used to test differences between repeated recordings of sEMG and VAS-score variables. Wilcoxon signed-rank test with a Bonferroni correction was used for post-hoc comparisons. Linear regression coefficients and Spearman’s ρ were used for correlation analyses. Significance level was set to p<0.05.

## Results

### Physiological responses

**Figure 2:**
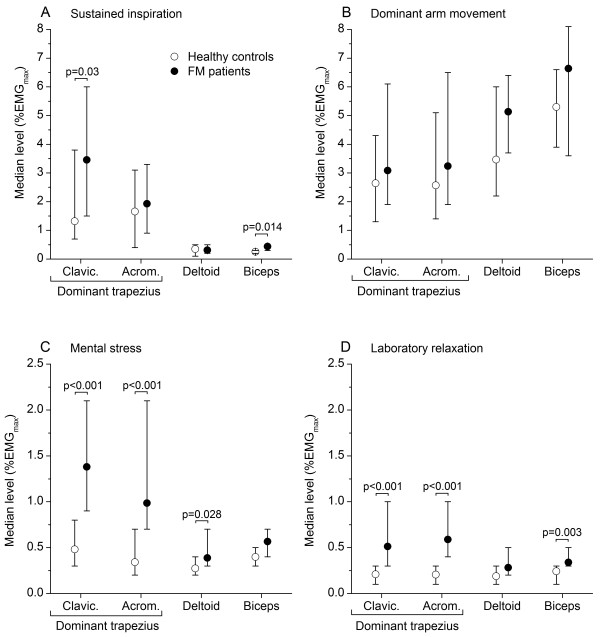
**sEMG activity during tests.** Median sEMG level (%EMG_max_) for clavicular and acromial fibers of dominant trapezius, middle deltoid, and biceps brachii during sympathetic activation, i.e., closing of epiglottis following deep inspiration (**A**), dominant arm movement (**B**), mental stress (**C**), and relaxation in the laboratory (**D**). Healthy controls (open circles) and fibromyalgia (FM) patients (filled circles) are represented by different symbols. Error bars indicate 95% CI of median.

Figure 2 shows sEMG responses in the laboratory tests. Clavicular trapezius sEMG activity was significantly higher for FM patients than HCs in sustained inspiration with breath holding (A). Both trapezius responses were markedly higher for FM patients vs. HCs in the mental stress test (C) and in Laboratory relaxation (D). sEMG rest time was correspondingly lower for FM patients (e.g., mental stress test: clavicular trapezius: 14 vs. 75%, p<0.011; acromial trapezius: 14 vs. 76%, p<0.001). Muscle activity during Laboratory relaxation was unchanged, comparing the first and last 5-min interval of the 30-min observation period. Trapezius sEMG activity of FM patients and HCs was similar in the test with dynamic arm movement (B). Statistically significant, but only moderately higher deltoid and biceps responses were observed for FM patients in several of the tests with higher trapezius responses. Downward adjustment of FM patient sEMG responses by 20% to compensate for putative submaximal effort in the calibration contractions did not alter the statistically significant differences of trapezius responses, comparing FM patients and HCs.

**Figure 3:**
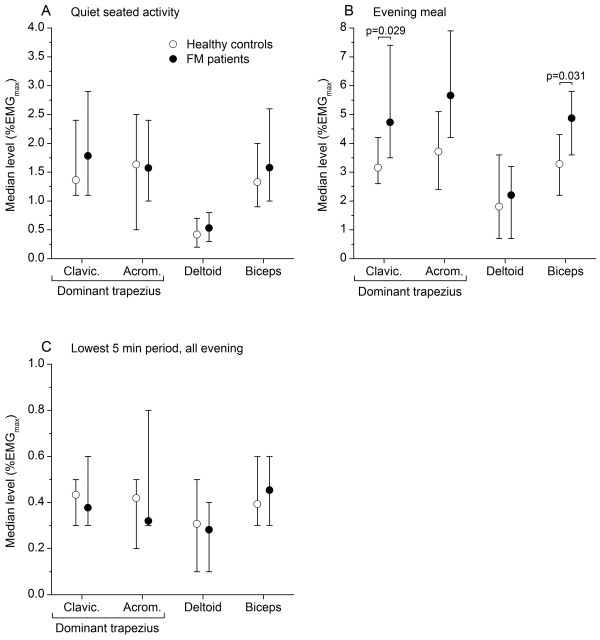
**sEMG activity during evening activities.** Median sEMG level (%EMG_max_) for clavicular and acromial fibers of dominant trapezius, middle deltoid, and biceps brachii during quiet seated activity at the patient hotel (**A**), evening meal (**B**), and the 5-min period with lowest sEMG activity during the evening (**C**). Healthy controls (open circles) and fibromyalgia (FM) patients (filled circles) are represented by different symbols. Error bars indicate 95% CI of median.

Figure 3 shows sEMG activity for quiet seated activity during the evening (A), for the evening meal (B), and for the 5-min period with lowest upper trapezius sEMG activity (C; always occurring during quiet seated activity). FM patients were not distinguished from HCs in quiet seated activity and lowest 5-min period, but showed higher trapezius and biceps activity during the evening meal. sEMG rest time during the evening did not distinguish FM patients from HCs (clavicular trapezius: 20 vs. 23%, acromial trapezius 30 vs. 23%; data not shown).

Table 3 presents HR responses. Repeated measures ANOVA showed a significant main effect of condition (F=41.1, df=1.7, p<0.001), but no main group effect (F=1.3, df=1, p=0.25), and no interaction between group and condition (F=2.3, df=1.7, p=0.12). Paired statistics showed elevated HR of FM patients in Laboratory relaxation relative to lowest 5-min during the evening (71 vs. 66 bpm), and further elevation in the mental stress test (76 bpm; Table 3). HR of HCs was unchanged in Laboratory relaxation vs. lowest 5-min period during the evening (67 vs. 65 bpm), and was elevated to the same level as for FM patients in the mental stress test (both groups at 76 bpm).

**Table 3:** Heart rate responses

	**Mental stress**	**Laboratory relaxation**	**Lowest 5 min, evening**
FM patients	76 (8), 58-89^b^	71 (8), 55-85^c^	66 (3), 52-78
Controls	76 (7), 64-88^d^	67 (8), 54-86	65 (3), 45-76
p^a^	0.89	0.06	0.35

Heart rate (bpm) during mental stress test, laboratory relaxation, and 5-min period with lowest heart rate during evening activities for fibromyalgia (FM) patients and healthy controls.

Values are mean (SD), range.

^a^Independent samples t-test; Paired samples t-test: ^b^different from Laboratory relaxation (p=0.001), and lowest 5 min period during evening (p<0.001); ^c^different from lowest 5 min period during evening (p=0.009); ^d^different from Laboratory relaxation (p<0.001) and lowest 5 min period during evening (p<0.001).

HRV analysis showed reduced variability for FM patients by the time domain variables SDNN (main group effect: F=8.51, df=1, p=0.006; no main effect of condition, no interaction between group and condition) and RMSSD (main group effect: F=5.05, df=1, p=0.03; main effect of condition: F=5.02, df=1.74, p=0.012; no interaction between group and condition). A non-significant tendency of elevated low-frequency HRV of FM patients in the stress test using time domain analysis (i.e., lower RMSSD) was very clear by frequency domain analysis (HF main condition effect: F=20,82, df=1.54, p<0.001; LF main condition effect: F=16.69, df=1.63, p<0.001; LF/HF ratio: F=13.43, df=1.68, p<0.001). Conversely, the frequency domain analysis did not show lower HRV for FM patients in the stress test.

### Subjective variables and the association to physiological responses

Shoulder/neck pain of FM patients was relatively high upon arrival in the laboratory, with a non-significant reduction after Laboratory relaxation (Table 4). The stress test provoked a statistically significant increase in shoulder/neck pain that stayed at the same elevated level till bedtime (38%, CI: 25–45). Low back pain of FM patients was lower than shoulder/neck pain (significant following Laboratory relaxation), and was unchanged following the stress test. Perceived general tension was higher for FM patients than HCs upon arrival and after Laboratory relaxation, and was elevated and equally high for the two groups following the stress test. FM patients and HCs scored similarly on perceived stressfulness of their day, and effort invested in the stress test.

**Table 4:** Subjective scores on Visual Analogue Scale (VAS) of pain (only fibromyalgia [FM] patients) and perceived general tension at arrival, after laboratory relaxation, and after mental stress test for FM patients and healthy controls

	**Arrival**	**Laboratory relaxation**	**Mental stress**	**p**^**a**^
Pain within FM group				
Shoulder/neck pain	28 (8–45)	22 (7–29)	39 (28–47)^c^	<0.001
Low back pain	12 (0–25)	7 (0–11)	7 (0–17)	0.09
p^b^	0.06	0.002	<0.001	
General tension				
FM patients	32 (13–42)	17 (7–25)	52 (37–58)^d^	<0.001
Controls	10 (5–24)^e^	5 (2–8)	40 (21–52)^f^	<0.001
p^b^	0.007	<0.001	0.13	

Values are median (95%CI).

^a^Friedman’s ANOVA; ^b^Wilcoxon signed rank test; ^c^different from Laboratory relaxation (p<0.001); ^d^different from arrival (p=0.03) and Laboratory relaxation (p<0.001); ^e^different from Laboratory relaxation (p<0.001) and mental stress (p<0.001); ^f^different from Laboratory relaxation (p<0.001).

Most items used to construct the indexes of musculoskeletal symptoms; muscle tension symptoms, non-musculoskeletal symptoms, and cognitive and psychological symptoms (cf. Methods) were scored significantly higher by FM patients vs. HC. The only significant correlation found between index scores and sEMG activity or HR was a negative correlation between musculoskeletal symptoms and clavicular trapezius activity for FM patients during the stress test (R=−.58, p=0.002).

## Discussion

To our knowledge, this is the first study to present data on muscle activity, HR, and HRV of FM patients in an unrestrained field setting to approximate daily living, together with responses to tests with stress exposure and instructed relaxation. Trapezius sEMG activity was higher for FM patients in the mental stress test and in sustained inspiration, which causes a sympathetic activation response [36,37]. FM patients further showed elevated upper trapezius activity in a laboratory rest situation and during the evening meal, the latter result potentially interesting as nutrient intake causes an increase in sympathetic activation [47,48].

FM patients were consistently distinguished from HCs by higher trapezius activity level in situations that trigger sympathetic activation (inspiratory breath holding, eating, mental stress test), valid for both trapezius electrode placements in most comparisons. A previous study with recording of single trapezius motor units indicated a stress-associated input to trapezius motoneurones [49], potentially representing a distinct pre-motor pathway. FM patients were not distinguished from HC by trapezius sEMG and HR in the 5 min period with trapezius activity at its lowest value in the field setting, when subject were watching TV or reading in their hotel room, or in the test with arm movement. These situations seem unlikely to trigger a sympathetic activation response. FM patients and HCs were further differentiated by trapezius activity in the laboratory rest period prior to the stress test. Elevated HR of FM patients in this period compared to the period with low trapezius activity in the evening may indicate a sympathetic activation response, an effect not observed for HCs. A possible explanation of this finding is that FM patients worry about the subsequent stress period and some form of anticipatory stress response is observed. Previous reports of elevated baseline activity and blunted responses of physiological variables with stress exposure, indicative of sympathetic activation, are consistent with this interpretation [50-52].

A previous study of physiological responses and pain development to sustained stress exposure, but using a different stress test, did not distinguish trapezius test responses of FM patients from HCs [20]. An important procedural difference between the two studies is that the previous study reported sEMG responses calibrated in absolute units (μV), due to the difficulty of achieving reliable reference contractions of forehead muscles. The large variation in sEMG_max_ (cf. Table 2) makes comparisons of sEMG using absolute unit calibration rather insensitive. Many of the statistically significant comparisons in the present material only showed statistical significance by one-tailed comparisons when recalculating results using absolute unit calibration. sEMG calibration by MVC or by sub-maximal reference contraction is however the preferred calibration procedure [26]. Stress exposure in the present study furthermore seems stronger as HCs showed markedly higher HR (ΔHR = 9 vs. 3 bpm; [53]) and shoulder/neck pain responses (17 vs. 3 VAS units after 30 min of stress exposure; [20]) than in the previous study. Indeterminate differences in test administration may cause differential sEMG responses if stress level in the test is low [19,20].

FM patients showed systematic lower sEMG responses during MVC (i.e., proportionally larger reduction in sEMG activity than the corresponding reduction in MVC force). This can be an error source or, alternatively, an interesting feature of muscles in this patient group. The reduction of sEMG amplitude in submaximal contractions at a set force level [54] suggests that low sEMG amplitude is a feature of FM patients. Low sEMG of FM patients may be due to disturbed muscle metabolism [10] or synchronization of active motor units [55]. Interstitial potassium is higher in patients with trapezius myalgia [56], potentially lowering muscle fiber action potentials and sEMG, and may occur also for FM patients.

It is debated whether FM represents the end point of a continuum from regional to generalized pain, or is a separate disorder [1,10,57,58]. Shoulder/neck pain was the dominant complaint of FM patients upon arrival in the laboratory. Stress exposure caused pain development in shoulder/neck, without influencing (much lower level) low back pain. FM patients were in this respect indistinguishable from patients with trapezius myalgia, favoring the integrated hypothesis on pathophysiological mechanisms. FM patients show muscle pathology [10,59] with clear similarities to muscle pathology in regional shoulder and neck pain [56,60,61]. The interest in muscular and autonomic responses of FM patients is rooted in evidence that peripheral components of the muscular and autonomic systems contribute to pain elicitation, additional to or integrated with CNS-based mechanisms [62]. Upper trapezius activity may not *per se* induce pain development, since trapezius activity in the stress test is similar to trapezius activity during the evening at the patient hotel and is at a level generally observed in sedentary living [63]. Trapezius activity may alternatively function as a marker of parallel pain-inducing activation responses, such as localized trigger point activity [8,36,64].

The FM patients in this study presented low levels of cortisol [31] and catecholamines [33], indicating down-regulation of target organs of both the hypothalamic-pituitary-adrenal axis and the sympathetic nervous system. This does not necessarily reflect the activity of central components of the sympathetic system; e.g., pituitary adrenocorticotropic hormone release was up-regulated in FM despite a relative depression of cortisol release [65]. Central components of the autonomic nervous system are clearly biased towards the sympathetic branch in FM, as indicated by low HRV [40,66], which was also observed for FM patients in this study. This may represent a basis for stress-associated upper trapezius activity.

The HRV variables in the frequency domain (LF, HF, LF/HF ratio; [66]), did not distinguish FM patients from HC, but showed a shift towards LF bias in the stress test, which was not observed with the time-domain variables (RMSSD showed a main effect of condition, no longer significant when FM patients and HC were analyzed separately). This illustrates that time-domain and frequency-domain variables describing HRV are not strictly comparable, although broadly equivalent [40,66]. The combined results show the expected effects of both group and condition on HRV responses: sympathetic bias of FM patients overall, and a shift towards sympathetic bias for both groups upon stress exposure.

The physiological results are consistent with the initial hypothesis; however, large inter-individual variation in responses and the possible influence of experimental conditions (e.g., stress level, experimental setting and circumstances of tests) suggest that the study should still be considered explorative and be replicated in similar study designs to fully understand stress-associated physiological responses of FM patients. It is conceivable that the mounting of recording equipment influence behavior and thus results. However, the long recording period and isolation from their everyday environment would argue against such an effect. Uncontrolled variation in adopted posture may contribute to the observed variation in sEMG results. Results on FM patient heterogeneity are reported in view of the interest in this aspect [24,52], but limited material implies low sensitivity of the analysis. A larger study base is required for differentiation of a FM patient population by index variables, but this is a demanding requirement for experimental studies with extensive physiological recording. A meta-analysis of several experimental studies may prove a future opportunity in this respect.

## Conclusion

FM patients in this study show clear evidence of disturbance of the autonomic system. Upper trapezius activity is similar to HC in unrestrained daily activities, but is enhanced in situations with performance-related and anticipative stress, presumably representing a provocation of the sympathetic nervous system. Generalized pain is a diagnostic criterion for FM; however, shoulder/neck pain dominates at arrival and is the dominant pain response to stress exposure. Similar, presumably peripheral mechanisms of pain elicitation may be associated with stressful experiences both for patients with FM and patients with regional pain in neck and shoulders.

## Competing interests

The authors have no conflicts of interest or competing financial interests to declare.

## Authors’ contributions

RHW participated in the design of the study, the collection of data, analysis and interpretation of data, and drafting the manuscript. PJM participated in the design of the study, the collection of data, analysis and interpretation of data, and drafting the manuscript. HWL participated in the design of the study, the collection of data, analysis and interpretation of data, and reviewed the manuscript for intellectual content. RR participated in the design of the study and reviewed the manuscript for intellectual content. UL participated in the design of the study, analysis and interpretation of data, and reviewed the manuscript for intellectual content. All authors read and approved the final manuscript.

## Pre-publication history

The pre-publication history for this paper can be accessed here:

http://www.biomedcentral.com/1471-2474/14/97/prepub
